# The effect of posterior subtenon methylprednisolone acetate in the refractory diabetic macular edema: a prospective nonrandomized interventional case series

**DOI:** 10.1186/1471-2415-6-15

**Published:** 2006-04-04

**Authors:** Alireza Javadzadeh

**Affiliations:** 1 Retina service, Department of Ophthalmology, Nikookari Hospital – Drug of applied research center, Tabriz University of Medical Sciences, Tabriz, Iran

## Abstract

**Background:**

To investigate the efficacy of posterior subtenon methylprednisolone acetate injection in treatment of refractory diffuse clinically significant diabetic macular edema (CSME).

**Methods:**

In a prospective, nonrandomized, interventional case series, 52 eyes were diagnosed with CSME and treated with at least two sessions of laser photocoagulation according to Early Treatment Diabetic Retinopathy Study guidelines. At least 3 months after laser therapy, eyes with a residual central macular thickness were offered posterior subtenon injection of 40 mg methylprednisolone acetate. Main outcome measures were visual acuity, macular thickness and intraocular pressure. Potential complications were monitored, including intraocular pressure response, cataract progression and scleral perforation.

**Results:**

Mean baseline visual acuity (in logMAR) improved significantly (*p *= 0.003) from 0.8 ± 0.36 to 0.6 ± 0.41 at 3 months. Mean foveal thickness decreased from 388 ± 78 μm at baseline to 231 ± 40 μm after 3 months (*p *< 0.0001). Visual acuity improvement in eyes with CSME with extrafoveal hard exudates was significant (*p *= 0.0001), but not significant in eyes with CSME with subfoveal hard exudates (*p =* 0.32). Intraocular pressure increased from 14.7 ± 2.0 mmHg (range, 12–18 mmHg) to a maximum value of 15.9 ± 2.1 mmHg (range, 12–20 mmHg) during the follow-up period. Complications in two eyes developed focal conjunctival necrosis at the site of injection.

**Conclusion:**

Posterior subtenon methylprednisolone acetate may improve early visual outcome in diffuse diabetic macular edema that fails to respond to conventional laser photocoagulation. Visual acuity improvement in eyes with CSME with extrafoveal hard exudates was significant; and this improvement is depends on location of hard exudates. Further study is needed to assess the long-term efficacy, safety, and retreatment.

## Background

Diabetic retinopathy is the major cause of blindness in the United States in patients younger than 50 years of age, and macular edema is the leading cause of visual impairment in diabetic patients [1]. As might be expected, the prevalence of macular edema is directly related to the overall severity of the retinopathy and instruction, ranging from 3% among eyes with mild non proliferative diabetic retinopathy (NPDR) to 38% among eyes with moderate to severe NPDR and 71% among eyes with proliferative diabetic retinopathy [2].

The Early Treatment Diabetic Retinopathy Study (EDTRS) showed that laser photocoagulation has significant benefit for the treatment of localized clinically significant macular edema [3]. However, some clinical features are associated with poorer visual acuity outcomes after photocoagulation: diffuse macular edema with center involved, diffuse fluorescein leakage, macular ischemia (extensive perifoveal capillary nonperfusion), hard exudates deposits in the foveola, marked cystoid macular edema [4]. Diffuse and prolonged edema may fail to respond to laser photocoagulation or may returns after initial improvement [5-7]. Substantial subgroups of patients with refractory macular edema prompted interest in other treatment modalities, including pars plana vitrectomy [8], medical therapy with protein kinase C inhibitors [9], intravitreal injection of corticosteroids [9-11] or a sustained release intravitreal corticosteroid implant [9].

Subtenon and retrobulbar injections of long acting corticosteroids have been used for treatment of cystoid macular edema (CME) secondary to uveitis [12,13] or intraocular surgeries [14]. Some studies showed acceptable results for intravitreal injection of triamcinolone acetonide, a corticosteroid suspension, in the treatment of uveitis and diabetic macular edema [9-11], [15,16]. Intravitreal injection has its potential complications and needs special setting to be done; also drug localization to the macula is important in obtaining maximum therapeutic effect [17].

Recent studies showed that trans-tenon's retrobulbar infusion and posterior subtenon injection of the corticosteroids are effective in treating posterior inflammation and macular edema associated with uveitis [12,18] and intraocular surgeries [19]. Because of localization of drug in the macula, posterior subtenon and retrobulbar injections are equally effective in the treatment of CME [17]. The main advantage of posterior subtenon steroid injection is a prolonged effect due to a maximal local concentration of the drug that causes minimal systemic side effects [20]. The aim of the present study is to assess the effects of subtenon injection of methylprednisolone acetate in the management of persistent and refractory clinically significant diabetic macular edema.

## Methods

In a prospective, nonrandomized, interventional case series 52 eyes of 52 diabetic individuals were diagnosed with clinically significant macular edema (CSME) according to EDTRS criteria. Approval for the study was obtained from the ethical committee of Tabriz University of Medical Sciences which is in compliance with the Helsinki Declaration. All patients received a thorough explanation of the study design and aims, and were provided with written informed consent. All patients were evaluated and treated by retina specialist. Inclusion criteria were patients with type II diabetes, persistent diffuse CSME 3 months after at least 2 sessions of macular laser photocoagulation, visual acuity loss, and leakage shown by fluorescein angiography (Imagenet 2000, Topcon TRC50IX, Topcon Corp, Japan). Eyes with history of glaucoma, cataract extraction, or other intraocular surgery were excluded from the study. Eyes with an epiretinal membrane, posterior hyaloid traction, ischemic maculopathy, and diabetic papillopathy were also excluded. The risks and benefits of the procedure were discussed with each patient before injection, and all patients provided written informed consent. Baseline parameters were documented including best corrected visual acuity, central macular thickness, intraocular pressure (IOP) and lenticular status. The best-corrected visual acuity was determined from the Early Treatment Diabetic Retinopathy Study (EDTRS) chart and calculated as logarithm of minimal angle of resolution (logMAR). Central macular thickness was measured by optical coherence tomography (OCT2; Zeiss-Humphrey inc., Dublin, Ca). Optical coherence tomography (OCT) was performed by acquiring six radial scans, 6 mm long, centered in the fovea, and then analyzed with retinal map protocol. Intraocular pressure was measured by applanation tonometer. Systemic condition of the patients were under control (blood glucose, blood pressure, and general condition), and they were receiving oral hypoglycemic agents or insulin for glycemic control.

All patients underwent posterior subtenon capsule injection of methylprednisolone acetate. Topical tetracaine was applied to the ocular surface. A cotton-tipped applicator soaked in tetracaine was then placed over the superotemporal quadrant for 2 minutes as the patient looked inferonasally. The methyl prednisolone acetate suspension was then shaken and 1 cc (40 mg) was drawn into a tuberculin syringe using a 25-gauge, 0.5-inch long needle. The upper eyelid was lifted, and at the patient looked inferonasally, the 25-gauge needle was used to penetrate the posterior subtenon space. Before injection of the methyl prednisolone acetate, the needle was moved from side to side to check that the sclera was not engaged in the needle tip. A 40 mg injection of methyl prednisolone was then injected in the posterior subtenon space.

Location of hard exudates, detected by slit-lamp examination with a 78-diopter indirect lens, fundus photography and fluorescein angiography; and all of the eyes divided into two groups. Group 1 including of 30 eyes having refractory diffuse CSME with extrafoveal hard exudates; and group 2 including of 22 eyes having refractory diffuse CSME with subfoveal hard exudates. Response to treatment was monitored by snellen visual acuity and OCT. IOP was recorded at every visit. Potential corticosteroid-induced and injection-related complications were also observed. Patients were observed 1 week, 1 and 3 months after injection.

An ANOVA test was used to compare the Values before and after treatment. Any differences showing a *p-*value of less than or equal to 0.05 were considered to be statistically significant.

## Results

The study included 52 eyes of 52 patients. Mean age ± SD of the patients was 55 ± 7.6 years (median, 55 years; range, 40 to 68 years). There were 25 males (48%) and 27 females (52%). There was no statistically significant difference in age and gender distribution of the patients. All patients were in the stage of non proliferative diabetic retinopathy (NPDR) and had at least two sessions of MPC. Mean follow-up time ± SD was 3.6 ± 0.9 months (median, 3 months; range 3–5 months). The mean logMAR visual acuity significantly improved from 0.8 ± 0.36 at baseline to 0.6 ± 0.42 at 1 week, 0.6 ± 0.43 at 1 month, and 0.6 ± 0.41 at 3 months (Table 1). The mean foveal thickness decreased from 388 ± 78 μm at baseline to 264 ± 61 μm at 1 week, 230 ± 39 μm at 1 month, and 231 ± 40 μm at 3 months [Table 1].

**Table 1:** Results of posterior subtenon methylprednisolone acetate injection for refractory diabetic macular edema.

	Mean ± SD				
		
	Before injection	After injection			*P*-Value*
			
		1 week	1 month	3 months	
VA (logMAR)	0.8 ± 0.36	0.6 ± 0.42	0.6 ± 0.43	0.6 ± 0.41	*0.003*
CMT (μm)	388 ± 78	264 ± 61	230 ± 39	231 ± 40	*0.0001*
IOP (mmHg)	14.7 ± 2.0	15.9 ± 2.1	15.7 ± 2.4	15.3 ± 2.3	*0.043*

CMT = Central macular thickness; IOP = intraocular pressure; logMAR = logarithm of minimal angle of resolution; VA = visual acuity; *p < 0.05 is statistically significant

Thirty eyes had CSME with extrafoveal hard exudates, and 22 eyes had CSME with subfoveal hard exudates. In eyes with CSME with extrafoveal hard exudates, visual acuity improvement was significant in follow-up interval (*p *< 0.0001); although was not significant in eyes with CSME with subfoveal hard exudates (*p *= 0.32); Evaluation of visual acuity and retinal thickness in two groups was explored. There were statistically significant differences between baseline logMAR visual acuity and follow-up intervals in eyes with CSME with extrafoveal hard exudates (*p *< 0001), although in eyes with CSME with Subfoveal hard exudates were not statistically significant (*p *= 0.32); moreover macular thickness in two groups significantly decreased during the follow-up period (*p *< 0.0001, *vs. p *< 0.0001 respectively) [Table 2].

**Table 2:** Results of posterior subtenon methylprednisolone acetate injection for refractory diabetic macular edema in two different groups.

	Mean ± SD				
		
	Before injection	After injection			*P*-Value*
			
		1 week	1 month	3 months	
Group 1(n = 30)					
VA (logMAR)	0.6 ± 0.27	0.3 ± 0.20	0.3 ± 0.21	0.3 ± 0.22	*0.0001*
CMT (μm)	367 ± 65	248 ± 44	222 ± 35	226 ± 40	*0.0001*
Group 2(n = 22)					
VA (logMAR)	1.1 ± 0.27	1.0 ± 0.27	1.0 ± 0.25	1.0 ± 0.25	*0.32*
CMT (μm)	417 ± 86	286 ± 76	241 ± 43	238 ± 39	*0.0001*

CMT = central macular thickness; Group 1 = eyes having refractory diffuse CSME with extrafoveal hard exudates; Group 2 = eyes having refractory diffuse CSME with subfoveal hard exudates; logMAR = logarithm of minimal angle of resolution; VA = visual acuity; *p < 0.05 is statistically significant

The mean baseline IOP was 14.7 ± 2.0 mmHg (range 12–18 mmHg), and at 1 week, 1 month, and 3 months after injection were 15.9 ± 2.1 mmHg (range 12–20), 15.7 ± 2.4 mmHg (range 12–20), and 15.3 ± 2.3 mmHg (range 12–18) respectively [Table 1].

In our study there were not any significant complications such as ocular hypertension (IOP>21), globe rupture, or cataract formation. Two patients developed focal conjunctival injection and necrosis over the injection site that was treated with antibiotic drops.

## Discussion

Macular edema is the most frequent cause of visual impairment in patients with NPDR [21]. Vascular permeability, resulting in the leakage of fluid and plasma constituents, such as lipoproteins into the retina, leads to thickening of the retina [22]. When thickening involves or threatens the center of the fovea, there is a higher risk of visual loss. The EDTRS investigators classified macular edema by its severity. It was defined as clinically significant macular edema (CSME), if any of the following features were present: thickening of the retina at or within 500 microns of the center of the macula, hard exudates at or within 500 microns of the center of the macula, if associated with thickening of the adjacent retina (not residual hard exudates remaining after the disappearance of retinal thickening), or a zone or zones of retinal thickening 1 disc area or larger, and ultimately any part of which in 1 disc diameter of the center of the macula [3]. Site of hard exudates is one of the important factors for visual prognosis after resolution of macular edema being either subfoveal or extrafoveal.

Corticosteroids have been used of their ability to inhibit the prostaglandin synthesis [10], and down regulate production of vascular endothelial growth factor (VEGF). Stabilization of blood retinal barrier (BRB) introduces a rationale for corticosteroid treatment of diabetic macular edema. Several studies showed the efficacy of intravitreal injection of corticosteroids in the management of diabetic macular edema [9-11]. Intravitreal injection is associated with rapid drug delivery to action site with maximal bioavailability, but has its complications. Recently trans-tenon's retrobulbar infusion and sub-tenon's injection of corticosteroids has been shown to be effective in the treatment of cystoid macular edema in patients with uveitis [12,15]. Subtenon injection of steroids in the treatment of uveitis was first reported in 1998 by Tanner et al [15]. It is a standard drug delivery method (with maximum concentration of drug in macula), which is used for treatment of chronic uveitis of posterior segment [13].

In retrospective, interventional case series, Chieh et al [23] treated 210 eyes with 1 or 4 mg of intravitreal triamcinolone acetonide for treatment of diffuse diabetic macular edema. They found a mean improvement in visual acuity from a median of 20/200 (mean logMAR, 0.92) at baseline to 20/80 (mean logMAR, 0.82) at 6 months. Mean intraocular pressure ± SD increased from 15.4 ± 3.4 mmHg to a maximal value of 20.4 ± 6.2 mmHg during the follow-up period. There were many complications, including culture-negative sterile endophthalmitis in six cases and cataract extraction in five eyes.

In prospective comparative, interventional case series, Jonas et al [11,24] studied 26 eyes injected with 25 mg of intravitreal triamcinolone acetonide for the treatment of diffuse diabetic macular edema. They showed that mean visual acuity ± SD improved significantly (*p *< 0.001) from 0.12 ± 0.08 at baseline to a maximum of 0.19 ± 0.14 during follow-up. Seventeen (81%) of 21 eyes with a follow-up of 1 month had improved visual acuity. Visual acuity did not change significantly in their control group. IOP also increased significantly (*p *< 0.001) and decreased significantly (p = 0.03) at the 5-month follow-up.

The main outcome measures of our study were best corrected visual acuity (by logMAR) and macular thickness (using OCT). Our results showed that, eyes had significant visual acuity improvement when compared with baseline as calculated by logMAR (*p *= 0.003), and also significant visual acuity improvement in eyes having CSME with extrafoveal hard exudates (p = 0.0001) but not significant in eyes having CSME with Subfoveal hard exudates (*p *= 0.32). In all eyes our results indicate that macular thickness significantly decreased between baseline and follow-up interval (*p *= 0.0001), and also significantly decreased in patients with CSME with extrafoveal and Subfoveal hard exudates (*p *= 0.0001 and, *p *= 0.0001) respectively [Table 2].

Ocular hypertension was not reported in this study. None of our patients had ocular complications of steroids, including development of cataract and glaucoma, prolonged healing of abrasions and epithelial defects [13]. Hemorrhage, globe perforation or perforation and extraocular muscle necrosis which are potential complications of injection itself was not reported in our study. In two patients developed focal conjunctival necrosis at the site of injection that was cured after 7–10 days of follow up with topical antibiotic.

Posterior subtenon methylprednisolone acetate may improve diabetic macular edema, but visual acuity improvement may depend on location of hard exudates. According to the results obtained, in addition to those factors excluded in the subject selection, it is important in the treatment of diabetic patients having CSME with hard exudates; the exact location of the hard exudates should be considered carefully.

## Conclusion

This method is associated with low potential complications related to intravitreal injections. However, long-term effects of posterior subtenon treatment are not clear. We believe further studies using a larger population with control will add valuable information to our results.

## Competing interests

The author(s) declares that he has no competing interest.

## Authors' contributions

AJ contributed to designing, implementing, analyzing and writing the manuscript.

## Pre-publication history

The pre-publication history for this paper can be accessed here:


